# Factors Associated With Mental Health Disorders Among University Students in France Confined During the COVID-19 Pandemic

**DOI:** 10.1001/jamanetworkopen.2020.25591

**Published:** 2020-10-23

**Authors:** Marielle Wathelet, Stéphane Duhem, Guillaume Vaiva, Thierry Baubet, Enguerrand Habran, Emilie Veerapa, Christophe Debien, Sylvie Molenda, Mathilde Horn, Pierre Grandgenèvre, Charles-Edouard Notredame, Fabien D’Hondt

**Affiliations:** 1 Department of Public Health, Centre Hospitalier Universitaire de Lille, Lille, France; 2 Fédération de Recherche en Psychiatrie et Santé Mentale des Hauts-de-France, Lille, France; 3 Centre National de Ressources et de Résilience Lille-Paris, Lille, France; 4 University Lille, Inserm, Centre Hospitalier Universitaire de Lille, CIC1403–Clinical Investigation Center, Lille, France; 5 Department of Psychiatry, Centre Hospitalier Universitaire de Lille, Lille, France; 6 University Lille, Inserm, Centre Hospitalier Universitaire de Lille, U1172–LilNCog–Lille Neuroscience and Cognition, Lille, France; 7 Department of Infant, Child and Adolescent Psychiatry, Avicenne Hospital, Assistance Publique–Hôpitaux de Paris, Sorbonne Paris Nord University, Centre de recherche en Epidémiologie et Santé des Populations, Bobigny, France; 8 Fonds Fédération Hospitalière de France Recherche et Innovation, Paris, France

## Abstract

**Importance:**

The coronavirus disease 2019 (COVID-19) pandemic and quarantine measures have raised concerns regarding their psychological effects on populations. Among the general population, university students appear to be particularly susceptible to experiencing mental health problems.

**Objectives:**

To measure the prevalence of self-reported mental health symptoms, to identify associated factors, and to assess care seeking among university students who experienced the COVID-19 quarantine in France.

**Design, Setting, and Participants:**

This survey study collected data from April 17 to May 4, 2020, from 69 054 students living in France during the COVID-19 quarantine. All French universities were asked to send an email to their students asking them to complete an online questionnaire. The targeted population was approximately 1 600 000 students.

**Exposure:**

Living in France during the COVID-19 quarantine.

**Main Outcomes and Measures:**

The rates of self-reported suicidal thoughts, severe distress, stress, anxiety, and depression were assessed using the 22-item Impact of Events Scale–Revised, the 10-item Perceived Stress Scale, the 20-item State-Trait Anxiety Inventory (State subscale), and the 13-item Beck Depression Inventory, respectively. Covariates were sociodemographic characteristics, precariousness indicators (ie, loss of income or poor quality housing), health-related data, information on the social environment, and media consumption. Data pertaining to care seeking were also collected. Multivariable logistic regression analyses were performed to identify risk factors.

**Results:**

A total of 69 054 students completed the survey (response rate, 4.3%). The median (interquartile range) age was 20 (18-22) years. The sample was mainly composed of women (50 251 [72.8%]) and first-year students (32 424 [47.0%]). The prevalence of suicidal thoughts, severe distress, high level of perceived stress, severe depression, and high level of anxiety were 11.4% (7891 students), 22.4% (15 463 students), 24.7% (17 093 students), 16.1% (11 133 students), and 27.5% (18 970 students), respectively, with 29 564 students (42.8%) reporting at least 1 outcome, among whom 3675 (12.4%) reported seeing a health professional. Among risk factors identified, reporting at least 1 mental health outcome was associated with female gender (odds ratio [OR], 2.10; 95% CI, 2.02-2.19; *P* < .001) or nonbinary gender (OR, 3.57; 95% CI, 2.99-4.27; *P* < .001), precariousness (loss of income: OR, 1.28; 95% CI, 1.22-1.33; *P* < .001; low-quality housing: OR, 2.30; 95% CI, 2.06-2.57; *P* < .001), history of psychiatric follow-up (OR, 3.28; 95% CI, 3.09-3.48; *P* < .001), symptoms compatible with COVID-19 (OR, 1.55; 95% CI, 1.49-1.61; *P* < .001), social isolation (weak sense of integration: OR, 3.63; 95% CI, 3.35-3.92; *P* < .001; low quality of social relations: OR, 2.62; 95% CI, 2.49-2.75; *P* < .001), and low quality of the information received (OR, 1.56; 95% CI, 1.49-1.64; *P* < .001).

**Conclusions and Relevance:**

The results of this survey study suggest a high prevalence of mental health issues among students who experienced quarantine, underlining the need to reinforce prevention, surveillance, and access to care.

## Key Points

**Question:**

What is the mental health state of university students in France who were confined during the coronavirus disease 2019 (COVID-19) pandemic, and what factors are associated with the development of mental health symptoms?

**Findings:**

This survey study of 69 054 students who experienced quarantine found high prevalence rates of severe self-reported mental health symptoms. Among risk factors identified, female or nonbinary gender, problems with income or housing, history of psychiatric follow-up, symptoms compatible with COVID-19, social isolation, and low quality of information received were associated with altered mental health.

**Meaning:**

The findings of this study suggest that students’ mental health is a public health issue that has become even more critical in the context of a pandemic, underlining the need to reinforce prevention, surveillance, and access to care.

## Teaser

This survey study measures the prevalence of self-reported mental health symptoms, identifies associated factors, and assesses care seeking among university students in France who experienced quarantine during the coronavirus disease 2019 (COVID-19) pandemic.

## Introduction

According to the US Centers for Disease Control and Prevention, quarantine consists of separating and restricting the movement of people who have been exposed to a contagious disease to check whether symptoms occur.^1^ Large-scale quarantine appears to be the most appropriate term to refer to a compulsory physical separation, including the restriction of movement, of populations who have been potentially exposed to a contagious disease.^2^ Large-scale quarantines are presented as strategies for reducing contact among people, and therefore, the spread of a contagion obliges, under pain of economic or criminal sanctions, a population to stay home.^1^

On January 30, 2020, the World Health Organization declared the global outbreak of the coronavirus disease 2019 (COVID-19) a public health emergency. Following several countries, such as China or Italy, the French government mandated a quarantine from March 17 to May 11, 2020. Citizens were asked to confine themselves to their homes, and unnecessary travel was prohibited. Based on lockdown experiences that were recorded in response to previous epidemics, a recent review of the literature concluded that quarantine measures could have negative psychological effects, including symptoms of posttraumatic stress, stress, anxiety, and depression.^3^

Among the general population, university students appear to be particularly susceptible to the negative impacts of quarantine.^4,5,6,7,8,9,10^ Indeed, before the COVID-19 outbreak, the mental health of young adults was already a global concern. In France, as in most high-income countries, suicide is the second-leading cause of death among individuals aged 15 to 25 years.^4^ In a 2016 national survey of 18 875 French university students, 37% of the participants declared having experienced an episode of depression, and 8% reported having suicidal thoughts in the past 12 months.^5^ Furthermore, the COVID-19 pandemic threatens to disrupt the provision of mental health services,^6^ and the most at-risk populations—primarily young individuals—are already the least likely to seek help.^7,8^ Finally, quarantine poses social and economic consequences, increasing the usual barriers to seeking care.^6^ During previous lockdowns, young adults (aged 16-24 years) had been shown to be especially at risk of mental health issues.^9^ During the initial stage of the COVID-19 epidemic, Wang et al^10^ found that students in China were at a greater risk of stress, anxiety, and depression in response to the COVID-19 outbreak than older adults.

This raises the question of the mental health burden of the pandemic on quarantined university students in France and of the factors associated with this. To our knowledge, this study is the first nationwide survey dedicated to students during the COVID-19 pandemic. It was conducted to establish the prevalence of self-reported suicidal thoughts and symptoms of distress, stress, depression, and anxiety; to identify potential factors associated with these concerns; and to assess the rate at which students sought mental health care.

## Methods

### Study Design and Population

The study used data from the repeated cross-sectional university-based survey Conséquences du contexte pandémique sur la santé mentale des étudiant (COSAMe), which we began conducting nationally on April 17, 2020. Measurement intervals are planned for a period of 1 year.

To reach students at each measurement interval, the French Ministry of Higher Education, Research, and Innovation asked 82 universities to send an email to their students (target population, approximately 1 600 000 students) offering them the opportunity to participate in the survey by completing an online questionnaire. Due to the heterogeneity of pandemic measures among countries, only students residing in France during the quarantine were included.

The first measurement interval took place between April 17 and May 4, 2020. During this period, the total number of confirmed cases of COVID-19 in France was nearly 170 000, and nearly 25 000 deaths were attributed to COVID-19.

This survey was examined by a French research ethics committee, the Comité de Protection des Personnes Ile de France VIII, before its initiation. Oral or written consent was not required for this study because responding to the survey was considered consent to participate. The survey was anonymous. No compensation was offered. This information was provided by email to all participants before giving them access to the questionnaire.

### Outcomes

We focused on the prevalence of the following 5 outcomes. First, we measured the prevalence of suicidal thoughts by asking participants whether they had experienced suicidal thoughts during the preceding month. Distress resulting from a traumatic life event was measured with the 22-item Impact of Events Scale–Revised (IES-R).^11^ Participants rate the extent to which each item applies to their experiences during the preceding 7 days, from 0 to 4. The total score ranges from 0 to 88. Thresholds established in the literature were as follows: normal distress level, 0 to 23; mild, 24 to 32; moderate, 33 to 36; and severe, greater than 36.^10^ Third, we used the 10-item Perceived Stress Scale (PSS-10) to measure stress. Respondents rate how often each item applies to their experiences during the preceding month, from 0 to 4. The total score ranges from 0 to 40.^12^ Thresholds used in the literature consider the stress level low for scores between 0 and 13; moderate, between 14 and 26; and high, greater than 26.^13,14^ We measured depression using the 13-item Beck Depression Inventory (BDI-13). Each question is rated from 0 to 3 to obtain a score ranging from 0 to 39.^15^ The authors of the BDI-13 recommend using the following classification: 0 to 3 (no depression), 4 to 7 (mild depression), 8 to 15 (moderate depression), and greater than 15 (severe depression).^16^ Anxiety was measured using the 20-item State-Trait Anxiety Inventory, State subscale (STAI Y-2).^17^ Participants rate items from 1 to 4 to obtain a score ranging from 20 to 80. The intensity of the state of anxiety increases with the score. Thresholds used in the French literature are as follows: low, less than 46; moderate, between 46 and 55; and high, greater than 55.^18^ Outcomes were the presence of severe self-reported symptoms, ie, the presence of suicidal thoughts or a high score on at least 1 scale (ie, IES-R >36; PSS-10 >26; BDI-13 >15; or STAI-Y2 >55).^10,11,12,13,14,15,16,17,18^

Regarding the factors associated with mental health outcomes, we considered sociodemographic, economic, social, and health-related factors classically associated with mental disorders as well as factors associated more directly with the pandemic context (eg, reduction in physical activity, isolation, economic consequences, level of exposure to a stressful event).^3,10^ Thus, covariates were as follows: (1) sociodemographic characteristics, including age, gender, year of study, being a foreign student, living area, department of residence (coded as an area with more than 50 deaths due to COVID-19 on March 29, 2020, or not); (2) precariousness indicators (ie, loss of income due to quarantine, housing quality [scale of 10, with 10 indicating the highest quality]); (3) health-related data (ie, history of psychiatric follow-up, symptoms consistent with COVID-19 since the beginning of the pandemic, and physical activity during the quarantine in minutes per day); (4) social relationship data (ie, feeling socially integrated before the quarantine [scale of 10, with 10 indicating highest social integration], having children, housing composition during the quarantine, concern for relatives’ health [scale of 10, with 10 indicating highest level of concern], quality of social relationships during the quarantine [scale of 10, with 10 indicating highest quality]; and (5) media and information data (consumption of media information related to the pandemic in minutes per day and quality of information received [scale of 10, with 10 indicating highest quality]). Participants also were asked to indicate whether they sought treatment for mental health reasons during the quarantine and, if yes, whether they accessed the university medical service or another health professional.

### Statistical Analysis

Only fully completed questionnaires were analyzed. First, we described the sample using medians with interquartile ranges (IQRs) for scores on measurement tools and quantitative covariates, given that they were mostly not normally distributed, and using numbers and percentages for the scores classified by level and other qualitative variables. Mental health outcome scores and distributions were described for the global sample and stratified by gender. Multivariable logistic regression analyses were performed to determine the potential risk factors of reporting at least 1 mental health outcome. Then, similar analyses were performed for each mental health outcome, ie, self-reported suicidal thoughts and severe self-reported symptoms of distress, stress, depression, and anxiety. All explanatory variables were included except age because of collinearity with the year of study. Associations between risk factors and outcomes are presented as odds ratios (ORs) and 95% CIs.

Data analysis was performed using R version 3.6.1 (R Project for Statistical Computing). The significance level was set at α = .05, and all tests were 2-tailed.

## Results

### Sample Characteristics

In total, 96 681 students opened the questionnaire. Among them, 69 054 (71.4%) completed it in its entirety (response rate, 4.3%; according to American Association of Public Opinion Reporting RR5, defined as completed interviews divided by the number of eligible individuals).^19^

The sample was mainly composed of women (50 251 [72.8%]), with 18 019 (26.1%) men and 784 (1.1%) nonbinary students (Table 1). The median (IQR) age was 20 (18-22) years. Less than half of the respondents (32 424 [47.0%]) were in their first year of study, whereas only 1436 (2.1%) were in their sixth year or later. Among the participants, 4282 (6.2%) were foreign students, and approximately one-quarter (18 599 [27.1%]) lived in areas with the highest rates of infection. During quarantine, 32 307 students (46.8%) lived in an urban area; 17 603 (25.5%), a semiurban area; and 19 144 (27.7%), a rural area.

**Table 1.** Factors Associated With Self-reported Mental Health Outcomes According to the Multivariable Logistic Regression Analysis

Characteristic	Students, No. (%)	≥1 Outcome			Suicidal thoughts			Symptoms of depression, BDI-13			Symptoms of distress, IES-R			Symptoms of stress, PSS-10			Symptoms of anxiety, STAI Y-2		
		aOR (95% CI)	*P *value		aOR (95% CI)	*P *value		aOR (95% CI)	*P *value		aOR (95% CI)	*P *value		aOR (95% CI)	*P *value		aOR (95% CI)	*P *value	
			Category	Overall		Category	Overall		Category	Overall		Category	Overall		Category	Overall		Category	Overall
**Demographic characteristics**																			
Gender																			
Male	18 019 (26.1)	1 [Reference]	NA	<.001	1 [Reference]	NA	<.001	1 [Reference]	NA	<.001	1 [Reference]	NA	<.001	1 [Reference]	NA	<.001	1 [Reference]	NA	<.001
Female	50 251 (72.8)	2.10 (2.02-2.19)	<.001		1.23 (1.16-1.31)	<.001		1.77 (1.67-1.88)	<.001		1.89 (1.80-1.99)	<.001		2.37 (2.26-2.50)	<.001		2.25 (2.14-2.36)	<.001	
Nonbinary	784 (1.1)	3.57 (2.99-4.27)	<.001		3.90 (3.30-4.61)	<.001		2.94 (2.47-3.49)	<.001		2.23 (1.89-2.63)	<.001		3.11 (2.65-3.65)	<.001		3.15 (2.64-3.67)	<.001	
Year of study																			
First	32 424 (47.0)	1 [Reference]	NA	<.001	1 [Reference]	NA	<.001	1 [Reference]	NA	<.001	1 [Reference]	NA	.002	1 [Reference]	NA	<.001	1 [Reference]	NA	<.001
Second or third	23 136 (33.5)	1.05 (1.01-1.10)	.007		0.97 (0.91-1.03)	.29		0.85 (0.81-0.90)	<.001		1.00 (0.96-1.05)	.80		1.14 (1.09-1.19)	<.001		1.15 (1.10-1.20)	<.001	
Fourth or fifth	12 058 (17.5)	1.02 (0.97-1.07)	.49		0.84 (0.77-0.90)	<.001		0.68 (0.63-0.73)	<.001		0.98 (0.93-1.04)	.52		1.08 (1.02-1.14)	.006		1.17 (1.11-1.24)	<.001	
Sixth or greater	1436 (2.1)	0.79 (0.69-0.89)	<.001		0.85 (0.70-1.02)	.09		0.34 (0.28-0.42)	<.001		0.77 (0.66-0.88)	<.001		0.71 (0.61-0.83)	<.001		0.91 (0.79-1.05)	.20	
Foreign student	4282 (6.2)	1.12 (1.04-1.20)	NA	.004	0.81 (0.72-0.90)	NA	<.001	0.96 (0.87-1.06)	NA	.45	1.57 (1.46-1.70)	NA	<.001	0.75 (0.69-0.81)	NA	<.001	1.02 (0.94-1.10)	NA	.61
Department of residence affected	18 599 (27.1)	1.09 (1.05-1.14)	NA	<.001	1.06 (1.00-1.12)	NA	.06	1.08 (1.02-1.14)	NA	.003	1.13 (1.08-1.18)	NA	<.001	1.13 (1.08-1.18)	NA	<.001	1.10 (1.05-1.14)	NA	<.001
Area																			
Urban	32 307 (46.8)	1 [Reference]	NA	.01	1 [Reference]	NA	<.001	1 [Reference]	NA	.08	1 [Reference]	NA	.04	1 [Reference]	NA	.41	1 [Reference]	NA	.10
Semiurban	17 603 (25.5)	1.03 (0.99-1.08)	.13		0.94 (0.88-1.00)	.05		1.01 (0.95-1.07)	.81		1.03 (0.98-1.09)	.17		1.03 (0.98-1.08)	.23		1.05 (1.00-1.10)	.03	
Rural	19 144 (27.7)	0.96 (0.92-1.00)	.08		0.88 (0.82-0.94)	<.001		0.94 (0.89-1.00)	.05		0.96 (0.92-1.01)	.18		1.00 (0.95-1.05)	.99		1.01 (0.96-1.06)	.61	
**Precariousness indicators**																			
Loss of income	15 120 (21.9)	1.28 (1.22-1.33)	NA	<.001	1.16 (1.09-1.23)	NA	<.001	1.33 (1.26-1.40)	NA	<.001	1.37 (1.31-1.43)	NA	<.001	1.17 (1.12-1.23)	NA	<.001	1.23 (1.18-1.29)	NA	<.001
Housing quality																			
High	56 831 (82.3)	1 [Reference	NA	<.001	1 [Reference]	NA	<.001	1 [Reference]	NA	<.001	1 [Reference]	NA	<.001	1 [Reference]	NA	<.001	1 [Reference]	NA	<.001
Medium	10 251 (14.8)	1.66 (1.58-1.75)	<.001		1.24 (1.16-1.33)	<.001		1.64 (1.55-1.74)	<.001		1.40 (1.33-1.48)	<.001		1.41 (1.34-1.49)	<.001		1.75 (1.67-1.85)	<.001	
Low	1972 (2.9)	2.30 (2.06-2.57)	<.001		1.38 (1.22-1.56)	<.001		2.47 (2.21-2.76)	<.001		1.87 (1.68-2.07)	<.001		1.93 (1.74-2.14)	<.001		2.23 (2.01-2.47)	<.001	
**Health-related data**																			
History of psychiatric follow-up	7114 (10.3)	3.28 (3.09-3.48)	NA	<.001	3.98 (3.74-4.23)	NA	<.001	3.02 (2.84-3.21)	NA	<.001	2.13 (2.02-2.26)	NA	<.001	2.52 (2.39-2.67)	NA	<.001	2.55 (2.41-2.70)	NA	<.001
Symptoms consistent with COVID-19	16 241 (23.5)	1.55 (1.49-1.61)	NA	<.001	1.55 (1.46-1.63)	NA	<.001	1.49 (1.42-1.56)	NA	<.001	1.55 (1.49-1.62)	NA	<.001	1.42 (1.36-1.48)	NA	<.001	1.43 (1.37-1.49)	NA	<.001
Duration of physical activity, min/d																			
≥60	24 748 (35.8)	1 [Reference]	NA	<.001	1 [Reference]	NA	<.001	1 [Reference]	NA	<.001	1 [Reference]	NA	<.001	1 [Reference]	NA	<.001	1 [Reference]	NA	<.001
30-59	19 192 (27.8)	1.13 (1.09-1.18)	<.001		1.07 (0.99-1.14)	.07		1.14 (1.07-1.21)	<.001		1.03 (0.98-1.08)	.25		1.16 (1.11-1.22)	<.001		1.15 (1.09-1.20)	<.001	
15-29	11 209 (16.2)	1.33 (1.27-1.40)	<.001		1.24 (1.15-1.34)	<.001		1.40 (1.31-1.51)	<.001		1.08 (1.02-1.15)	.006		1.40 (1.32-1.48)	<.001		1.36 (1.29-1.44)	<.001	
<15	13 905 (20.1)	1.50 (1.43-1.58)	<.001		1.30 (1.22-1.40)	<.001		1.84 (1.73-1.96)	<.001		1.12 (1.06-1.19)	<.001		1.61 (1.53-1.70)	<.001		1.61 (1.53-1.70)	<.001	
**Social ties**																			
Having children, yes vs no	1212 (1.8)	0.68 (0.60-0.78)	NA	<.001	0.55 (0.43-0.69)	NA	<.001	0.61 (0.50-0.74)	NA	<.001	0.94 (0.82-1.09)	NA	.44	0.47 (0.40-0.56)	NA	<.001	0.75 (0.65-0.86)	NA	<.001
Housing arrangement																			
Living with family	56 480 (81.8)	1 [Reference]	NA	<.001	1 [Reference]	NA	<.001	1 [Reference]	NA	<.001	1 [Reference]	NA	<.001	1 [Reference]	NA	.009	1 [Reference]	NA	.001
Living alone	8651 (12.5)	1.12 (1.06-1.19)	<.001		1.33 (1.23-1.43)	<.001		1.20 (1.12-1.29)	<.001		1.19 (1.12-1.26)	<.001		1.04 (0.97-1.10)	.26		1.06 (1.00-1.13)	.05	
Living with roommates	2990 (4.3)	1.23 (1.13-1.35)	<.001		1.44 (1.28-1.62)	<.001		1.13 (1.01-1.26)	.04		1.25 (1.14-1.38)	<.001		1.16 (1.06-1.28)	.002		1.18 (1.07-1.29)	<.001	
Other	933 (1.4)	1.18 (1.01-1.36)	.03		1.70 (1.41-2.05)	<.001		1.32 (1.10-1.58)	.003		1.22 (1.04-1.43)	.01		1.12 (0.95-1.31)	.16		1.16 (0.99-1.35)	.06	
Feelings of integration																			
High	45 306 (65.6)	1 [Reference]	NA	<.001	1 [Reference]	NA	<.001	1 [Reference]	NA	<.001	1 [Reference]	NA	<.001	1 [Reference]	NA	<.001	1 [Reference]	NA	<.001
Medium	19 433 (28.1)	2.11 (2.03-2.20)	<.001		2.61 (2.47-2.76)	<.001		2.83 (2.69-2.97)	<.001		1.43 (1.37-1.49)	<.001		1.96 (1.88-2.04)	<.001		1.99 (1.91-2.07)	<.001	
Low	4315 (6.2)	3.63 (3.35-3.92)	<.001		5.05 (4.65-5.48)	<.001		6.09 (5.64-6.58)	<.001		1.67 (1.54-1.80)	<.001		3.02 (2.81-3.25)	<.001		2.81 (2.61-3.03)	<.001	
Concern for relatives’ health																			
Low	9072 (13.1)	1 [Reference]	NA	<.001	1 [Reference]	NA	<.001	1 [Reference]	NA	<.001	1 [Reference]	NA	<.001	1 [Reference]	NA	<.001	1 [Reference]	NA	<.001
Medium	19 612 (28.4)	1.10 (1.04-1.17)	.001		0.81 (0.75-0.88)	<.001		0.94 (0.86-1.02)	.13		1.20 (1.11-1.30)	<.001		1.12 (1.04-1.20)	.002		1.21 (1.13-1.31)	<.001	
High	40 370 (58.5)	2.12 (2.00-2.24)	<.001		0.87 (0.81-0.94)	<.001		1.53 (1.42-1.65)	<.001		2.53 (2.36-2.72)	<.001		1.87 (1.76-2.00)	<.001		2.57 (2.41-2.75)	<.001	
Quality of social ties																			
High	30 594 (44.3)	1 [Reference]	NA	<.001	1 [Reference]	NA	<.001	1 [Reference]	NA	<.001	1 [Reference]	NA	<.001	1 [Reference]	NA	<.001	1 [Reference]	NA	<.001
Medium	26 745 (38.7)	1.59 (1.53-1.65)	<.001		1.40 (1.31-1.48)	<.001		1.70 (1.61-1.79)	<.001		1.42 (1.36-1.48)	<.001		1.50 (1.44-1.57)	<.001		1.74 (1.67-1.81)	<.001	
Low	11 715 (17.0)	2.62 (2.49-2.75)	<.001		1.95 (1.82-2.09)	<.001		2.96 (2.78-3.15)	<.001		2.12 (2.01-2.24)	<.001		2.37 (2.24-2.50)	<.001		2.83 (2.69-2.99)	<.001	
**Media and information**																			
Time spent consulting information, min/d																			
<15	26 240 (38.3)	1 [Reference]	NA	<.001	1 [Reference]	NA	.04	1 [Reference]	NA	<.001	1 [Reference]	NA	<.001	1 [Reference]	NA	<.001	1 [Reference]	NA	<.001
15-29	11 583 (16.8)	1.18 (1.12-1.24)	<.001		0.99 (0.92-1.07)	.82		1.10 (1.03-1.18)	.006		1.27 (1.19-1.34)	<.001		1.17 (1.10-1.23)	<.001		1.26 (1.19-1.33)	<.001	
30-59	14 064 (20.4)	1.25 (1.19-1.31)	<.001		0.98 (0.91-1.05)	.60		1.12 (1.05-1.20)	<.001		1.41 (1.34-1.49)	<.001		1.20 (1.14-1.26)	<.001		1.35 (1.29-1.43)	<.001	
60-119	11 277 (16.3)	1.47 (1.40-1.55)	<.001		1.01 (0.93-1.09)	.86		1.29 (1.21-1.38)	<.001		1.60 (1.51-1.70)	<.001		1.41 (1.33-1.49)	<.001		1.68 (1.59-1.78)	<.001	
≥120	5710 (8.3)	1.89 (1.77-2.02)	<.001		1.14 (1.04-1.26)	.005		1.68 (1.55-1.82)	<.001		1.96 (1.83-2.09)	<.001		1.63 (1.52-1.74)	<.001		2.08 (1.94-2.23)	<.001	
Quality of information received																			
High	24 226 (35.1)	1 [Reference]	NA	<.001	1 [Reference]	NA	<.001	1 [Reference]	NA	<.001	1 [Reference]	NA	<.001	1 [Reference]	NA	<.001	1 [Reference]	NA	<.001
Medium	32 763 (47.4)	1.27 (1.22-1.32)	<.001		1.05 (0.99-1.12)	.08		1.23 (1.16-1.29)	<.001		1.25 (1.20-1.31)	<.001		1.25 (1.20-1.30)	<.001		1.36 (1.30-1.42)	<.001	
Low	12 065 (17.5)	1.56 (1.49-1.64)	<.001		1.21 (1.12-1.30)	<.001		1.45 (1.35-1.55)	<.001		1.56 (1.48-1.65)	<.001		1.54 (1.46-1.63)	<.001		1.57 (1.48-1.66)	<.001	

Abbreviations: aOR, adjusted odds ratio; BDI-13, 13-item Beck Depression Inventory; IES-R, Impact of Events Scale–Revised; NA, not applicable; PSS-10, 10-item Perceived Stress Scale; STAI Y-2, 20-item State-Trait Anxiety Inventory, State subscale.

Concerning precariousness indicators, the median (IQR) score indexing the quality of their housing was 8 (7-10) of 10. Overall, 15 120 students (21.9%) declared a loss of income because of the quarantine.

Health information was as follows: 7114 respondents (10.3%) reported a history of psychiatric follow-up, 16 241 (23.5%) reported having experienced symptoms consistent with COVID-19, and participants declared a median (IQR) duration of physical activity of 40 (17-70) minutes per day during quarantine.

Regarding social ties, 1212 students (1.8%) had children. During the quarantine, most students (56 480 [81.8%]) lived with relatives, 8651 (12.5%) lived alone, and 2990 (4.3%) lived with roommates. The median (IQR) score indexing the feeling of integration before quarantine was 7 (6-8) of 10. Participants rated their worries concerning relatives’ health a median (IQR) score of 7 (5-8) of 10, and the quality of the social ties they maintained during quarantine was rated a median (IQR) score of 6 (4-8) of 10.

Finally, participants rated the quality of the information related to COVID-19 and quarantine they received a median (IQR) score of 6 (4-7) of 10. They reported spending a median (IQR) of 20 (7-50) minutes a day accessing COVID-19 and quarantine information.

### Mental Health Outcomes

The prevalence rates of self-reported suicidal thoughts and severe distress (IES-R), perceived stress (PSS-10), depression (BDI-13), and anxiety (STAI-Y2) were 11.4% (7891 students), 22.4% (15 463 students), 24.7% (17 093 students), 16.1% (11 133 students), and 27.5% (18 970 students), respectively. A total of 29 564 students (42.8%) reported at least 1 outcome. Self-reported symptoms stratified by gender are presented in Table 2.

**Table 2.** Self-reported Mental Health Outcomes According to Gender

Outcome	Students, No. (%)				*P* value
	All (N = 69 054)	Male (n = 18 019)	Female (n = 50 251)	Nonbinary (n = 784)	
≥1 outcome	29 564 (42.8)	5534 (30.7)	23 467 (46.7)	563 (71.8)	<.001
Suicidal thoughts	7891 (11.4)	1783 (9.9)	5745 (11.4)	363 (46.3)	<.001
IES-R score					
Median (IQR)	20 (10-35)	14 (6-27)	22 (12-36)	28 (13-44)	<.001
Normal	39 173 (56.7)	12 493 (69.3)	26 343 (52.4)	337 (43.0)	<.001
Mild	10 477 (15.2)	2145 (11.9)	8220 (16.4)	112 (14.3)	
Moderate	3941 (5.7)	751 (4.2)	3131 (6.2)	59 (7.5)	
Severe	15 463 (22.4)	2630 (14.6)	12 557 (25.0)	276 (35.2)	
PSS-10 score					
Median (IQR)	20 (14-26)	17 (11-23)	22 (16-27)	26 (20-31)	<.001
Low	15 754 (22.8)	6559 (36.4)	9131 (18.2)	64 (8.2)	<.001
Moderate	36 207 (52.4)	8820 (48.9)	27 039 (53.8)	348 (44.4)	
High	17 093 (24.7)	2640 (14.6)	14 081 (28.0)	372 (47.4)	
BDI-13 score					
Median (IQR)	7 (3-13)	6 (2-11)	8 (4-13)	13 (8-20)	<.001
Normal	17 625 (25.5)	6326 (35.1)	11 241 (22.4)	58 (7.4)	<.001
Mild	17 356 (25.1)	4621 (25.6)	12 619 (25.1)	116 (14.8)	
Moderate	22 940 (33.2)	4988 (27.7)	17 674 (35.2)	278 (35.5)	
Severe	11 133 (16.1)	2084 (11.6)	8717 (17.3)	332 (42.3)	
STAI Y-2 score					
Median (IQR)	45 (34-57)	38 (29-51)	47 (36-58)	55 (44-65)	<.001
Low	35 456 (51.3)	11 877 (65.9)	23 354 (46.5)	225 (28.7)	<.001
Moderate	14 628 (21.2)	3061 (17.0)	11 400 (22.7)	167 (21.3)	
High	18 970 (27.5)	3081 (17.1)	15 497 (30.8)	392 (50.0)	

Abbreviations: BDI-13, 13-item Beck Depression Inventory; IES-R, Impact of Events Scale–Revised; IQR, interquartile range; PSS-10, 10-item Perceived Stress Scale; STAI Y-2, 20-item State-Trait Anxiety Inventory, State subscale.

### Factors Associated With Mental Health Outcomes

#### Sociodemographic Characteristics

Multivariate analyses are presented in Table 1. Reporting at least 1 outcome was associated with female gender (OR, 2.10; 95% CI, 2.02-2.19; *P* < .001) and nonbinary gender (OR, 3.57; 95% CI, 2.99-4.27; *P* < .001), being a foreign student (OR, 1.12; 95% CI, 1.04-1.20; *P* = .004), living in areas with the highest rates of infection (OR, 1.09; 95% CI, 1.05-1.14; *P* < .001), and year of study (for sixth year vs first year: OR, 0.79; 95% CI, 0.69-0.89; *P* < .001).

Concerning detailed outcomes, female and nonbinary genders were associated with an increased risk for all outcomes. Students just beginning their university education were at increased risk of self-reported suicidal thoughts, severe depression, or severe distress; the more advanced the students were, the less at risk. Concerning perceived stress and anxiety, an increased risk was identified among students in their second or third year and fourth or fifth year, whereas students in their sixth year or higher appeared less likely to report these symptoms. Being a foreign student was associated with a lower likelihood of self-reported suicidal thoughts and perceived stress but an increased risk of distress. For students living in the worst-hit areas, a slightly increased risk was observed for all outcomes except suicidal thoughts. Finally, living in rural areas was associated with a lower likelihood of self-reported suicidal thoughts and severe distress than living in an urban area.

#### Precariousness Indicators

Students who experienced a loss of income were at higher risk of reporting at least 1 mental health outcome compared with those who did not (OR, 1.28; 95% CI, 1.22-1.33; *P* < .001), and the lower the quality of the accommodation was, the higher the risk of experiencing mental health symptoms (medium quality vs high quality: OR, 1.66; 95% CI, 1.58-1.75; *P* < .001; low quality vs high quality: OR, 2.30; 95% CI, 2.06-2.57; *P* < .001). Similar results were found for all outcomes.

#### Health-Related Data

Reporting at least 1 outcome was associated with history of psychiatric follow-up (OR, 3.28; 95% CI, 3.09-3.48; *P* < .001), symptoms consistent with COVID-19 (OR, 1.55; 95% CI, 1.49-1.61; *P* < .001), and physical activity. More frequent physical activity was associated with less severe self-reported symptoms (30-59 min/d vs 60 min/d: OR, 1.13; 95% CI, 1.09-1.18; *P* < .001; 15-29 min/d vs 60 min/d: OR, 1.33; 95% CI, 1.27-1.40; *P* < .001; <15 min/d vs 60 min/d: OR, 1.50; 95% CI, 1.43-1.58; *P* < .001). Similar results were found for all outcomes.

#### Social Ties

Having children was associated with a lower likelihood of reporting at least 1 outcome (OR, 0.68; 95% CI, 0.60-0.78; *P* < .001). Housing conditions were also associated with impaired mental health: globally, not living with family was associated with a higher likelihood of reporting at least 1 mental health outcome (living alone: OR, 1.12; 95% CI, 1.06-1.18; *P* < .001; living with roommates: OR, 1.23; 95% CI, 1.13-1.35; *P* < .001). Furthermore, participants reporting weaker feelings of social integration before quarantine had a higher risk of experiencing mental health symptoms (medium vs high social integration: OR, 2.11; 95% CI, 2.03-2.20; *P* < .001; low vs high social integration: OR, 3.63; 95% CI, 3.35-3.92; *P* < .001). Conversely, worrying about the health of relatives was associated with a higher risk of reporting an outcome (medium vs low level of worry: OR, 1.10; 95% CI, 1.04-1.17; *P* = .001; high vs low level of worry: OR, 2.12; 95% CI, 2.00-2.24; *P* < .001). Finally, the risk of reporting at least 1 outcome increased as the quality of social bonds during quarantine decreased (medium vs high quality: OR, 1.59; 95% CI, 1.53-1.65; *P* < .001; low vs high quality: OR, 2.62; 95% CI, 2.49-2.75; *P* < .001).

Concerning detailed outcomes, having children was associated with lower risk of all outcomes except severe distress. A higher level of worry was associated with an increased risk of all outcomes except suicidal thoughts; students reporting medium or high levels of worry had a lower risk of reporting suicidal thoughts. For other variables (housing conditions, feeling of integration, and quality of the social bonds), results were globally similar for each outcome.

#### Information and Media

The more time students spent consulting the news, the more likely they were to report at least 1 outcome (15-29 min/d vs <15 min/d: OR, 1.18; 95% CI, 1.12-1.24; *P* < .001; 30-59 min/d vs <15 min/d: OR, 1.25; 95% CI, 1.19-1.31; *P* < .001; 60-119 min/d vs <15 min/d: OR, 1.47; 95% CI, 1.40-1.55; *P* < .001; ≥120 min/d vs <15 min/d: OR, 1.89; 95% CI, 1.77-2.02; *P* < .001). Finally, lower quality information was associated with reporting at least 1 outcome (medium vs high quality: OR, 1.27; 95% CI, 1.22-1.32; *P* < .001; low vs high quality: OR, 1.56; 95% CI, 1.49-1.64; *P* < .001). Results were globally similar for all outcomes.

### Use of Mental Health Care

Among all students, 4682 (6.8%) reported seeing a professional for mental health reasons, and 1037 (1.5%) reported having requested the university health service. Of the 29 564 students with at least 1 outcome, 3675 (12.4%) consulted a mental health professional, and 810 (2.7%) used the university service.

## Discussion

This large nationwide study revealed a high prevalence of self-reported suicidal thoughts and severe self-reported distress, depression, anxiety, and stress among quarantined students. Among the identified risk factors, having female or nonbinary gender, experiencing loss of income, having poor quality housing, having a history of psychiatric follow-up, having symptoms compatible with COVID-19, having a low level of physical activity, not living with family, having a weak sense of integration, having a low quality of social relations, and receiving low-quality information were associated with all mental health issues. Use of mental health care services was remarkably low during the lockdown.

The rates of mental health disorders are consistent with preliminary data recently reported among the Chinese general population during the initial stage of the COVID-19 epidemic,^10^ and they appear to be higher than the estimates obtained among students before quarantine. Indeed, the results of a national survey conducted among French students in 2016 found that 15% reported a depressive episode in the last 4 weeks.^5^ Here, we found that 16.1% reported severe symptoms of depression. The proportion reached 74.4% when considering mild to severe self-reported symptoms. The same survey found that 8% of students had suicidal thoughts during the last 12 months.^5^ In the present study, the prevalence was at 11.4% during a shorter period of only 1 month. In France, the European Study of the Epidemiology of Mental Disorders/Mental Health Disability population-based study^20^ estimated the prevalence of anxiety disorder in the past 12 months at 9.8% and the prevalence of posttraumatic stress (at any given time during the past month) at 0.9%. In this study, 27.5% of students reported a high level of anxiety at the moment of the inquiry, and 22.4% reported severe distress.

Risk factors identified by the present study are also consistent with those reported in the literature on quarantines. The review by Brooks et al^3^ pointed out that female gender, history of psychiatric illness, experiencing physical symptoms, concerns about relatives’ health, reduced social contact, lack of information, and financial loss were all associated with mental health disorders. In the context of the COVID-19 epidemic, Wang et al^10^ showed that satisfaction with information received and high levels of concern about family members getting COVID-19 were significantly associated with high levels of distress, stress, anxiety, and depression.

Finally, the use of mental health care appears to have decreased during the quarantine. For example, before quarantine, people aged 16 to 24 years with high levels of depression or anxiety obtained professional help in 18% to 34% of cases.^21,22^ Here, students with self-reported suicidal thoughts or severe anxiety, stress, distress, or depression sought mental health care only 12.4% of the time. This result is consistent with those from previous quarantines, showing that access to regular medical care and prescriptions was problematic.^3^ Such a situational barrier may be particularly detrimental for young people, who are known to poorly access care at baseline.^22^ Given that access to care is known as a major pillar of prevention,^23,24^ it further reinforces the recent call by Kannarkat et al^6^ for the mobilization and development of telepsychiatry.

### Limitations

Some limitations should be considered in the interpretation of these results. First, although the number of respondents is large, it represents 4.3% of students contacted, and self-selection bias may have altered the results. There was notably an overrepresentation of women. Nevertheless, this problem is encountered in all large epidemiologic studies.^25^ This justified the choice to stratify the prevalence results by gender. This overrepresentation was considered in the multivariate analysis; gender was included as a covariate. It has also been shown that a low response rate in epidemiological surveys only marginally affects prevalence and association measures. When the studies focus on stigmatized behaviors or diseases, it is difficult to recruit participants who are affected by those behaviors or diseases.^25,26^

These results must be considered in the context of the acute phase of the pandemic, while people were quarantined at home. This appears particularly important for the interpretation of the high rate of severe self-reported distress symptoms evidenced here. Posttraumatic stress disorder is known to be a potential consequence of major disasters. In the study by Wang et al,^10^ 53.8% of participants reported moderate or severe symptoms of distress during the initial stage of the COVID-19 pandemic. Using the same threshold for the IES-R (ie, ≥33), we found 28.1%. These proportions are particularly high compared with the usual proportions of posttraumatic stress disorder in these countries (ie, <1%).^20,27^ Therefore, it appears to support the concern expressed by Dutheil et al^28^ about the risk of posttraumatic stress disorder as the second tsunami of the COVID-19 pandemic.^28^ However, the IES-R is not a diagnostic tool, and diagnostic criteria for posttraumatic stress disorder require symptoms to last for more than 1 month.^29^ Therefore, it would be important to determine whether posttraumatic stress disorder develops later, which is what is planned in the COSAMe study.

Furthermore, the present study cannot establish the direct association between the high rates of severe self-reported mental health symptoms and the COVID-19 pandemic and quarantine. However, high rates were also found during previous quarantines and in China during the COVID-19 pandemic.^3,10^

## Conclusions

In this study, university students in France reported high rates of suicidal thoughts and severe symptoms of distress, depression, anxiety, and perceived stress while quarantined during the COVID-19 pandemic. Protecting the mental health of students is a public health issue that appears even more critical in the context of a pandemic. The results suggest that special attention must be paid to women and nonbinary students as well as students with a history of psychiatric follow-up. It also appears important to maintain contact with students, to ensure they have good quality housing conditions, to provide for their basic needs, to allow them to maintain physical activity and social ties, and to give them adequate information. Measures promoting access to care should be encouraged.
